# Family physicians collaborating for health system integration: a scoping review

**DOI:** 10.1186/s12913-023-09063-w

**Published:** 2023-01-23

**Authors:** Colleen Grady, Han Han, Da Hye Kim, Angela M. Coderre-Ball, Nadia Alam

**Affiliations:** 1 Centre for Studies in Primary Care, Department of Family Medicine, Queen’s University, 220 Bagot Street, Kingston, ON K7L 5E9 Canada; 2 School of Medicine, Queen’s University, Kingston, Canada; 3 Halton Healthcare, Toronto, Canada; 4 DFCM and IHPME, University of Toronto, Toronto, Canada

**Keywords:** Family physician, Integrated care, Inter-organizational collaboration, Structure, Processes

## Abstract

**Background:**

In Canada, Ontario Health Teams (OHTs) are a new model for integrated healthcare. Core to OHTs are family physicians (FPs) and their ability to collaborate with other FPs and healthcare providers. Whereas the factors for intra-organizational collaboration have been well-studied, inter-organizational collaboration between FPs and other healthcare organizations as an integrated care network, are less understood. This paper aims to explore the structural factors, processes, and theoretical frameworks that support FPs’ collaboration for integrated healthcare.

**Methods:**

A scoping review was undertaken based on Joanna Briggs Institute (JBI) methodology for scoping review and using the Preferred Reporting Items for Systematic Review and Meta-Analysis for Scoping Review (PRISMA_ScR) checklist. A search for academic and relevant grey literature published between 2000–2021 was conducted across databases (MEDLINE, EMBASE, EBSCOhost).Thematic analysis was used to identify the key findings of the selected studies.

**Results:**

Thirty-two studies were included as eligible for this review. Three structural components were identified as critical to FPs’ successful participation in inter-organizational partnerships: (1) shared vision/values, (2) leadership by FPs, and (3) defined decision-making procedures. Also, three processes were identified: (1) effective communication, (2) a collective sense of motivation for change, and (3) relationships built on trust. Three theoretical frameworks provided insight into collaborative initiatives: (1) Social Identity Approach, (2) framework of interprofessional collaboration, and (3) competing values framework.

**Conclusion:**

FPs hold unique positions in healthcare and this review is the first to synthesize the best evidence for building collaborations between FPs and other healthcare sectors. These findings will inform collaboration strategies for healthcare integration, including with OHTs.

**Supplementary Information:**

The online version contains supplementary material available at 10.1186/s12913-023-09063-w.

## Introduction

There is significant evidence that improved patient and health system outcomes are achievable when primary care is central to the health system at large [1] but there are challenges to involving family physicians (FPs) in the development of an integrated health system in Canada [2–4]. FPs are intimately knowledgeable about the health system but often isolated from broader health system structures due to the independent nature of their work [5, 6], limiting their ability to contribute to change. Additionally, the best practices for tapping into the wisdom of FPs remains largely unknown as are the processes to engage them [2]. The delivery of quality patient care will continue to face numerous challenges unless reform efforts include FPs for meaningful and productive dialogue and most importantly, evidence-informed change [7].

Globally, health systems are experiencing increased pressure to provide care in the face of increasing prevalence of chronic disease and decreasing resources [8, 9]. To combat these challenges, we need a fully integrated health system [10]. WHO defines integrated care as “bringing together inputs, delivery, management and organization of services related to diagnosis, treatment, care, rehabilitation and health promotion. Integration is a means to improve services in relation to access, quality, user satisfaction and efficiency” ([11], p.7). In Canada, the province of Ontario introduced Local Health Integrated Networks (LHINs) in 2004, tasked with planning, integrating, and distributing government health funding at the regional level [12]. More recently, Ontario Health Teams (OHTs, groups of providers and organizations) were tasked with being clinically and fiscally accountable for delivering a full and coordinated continuum of care within a geographic region [13].

### Tapping into the knowledge of family physicians

Given their unique position in the healthcare system and the role they play in patient care, FPs’ role in the co-design of a more effective system of care is incontrovertible [14]. However, their participation is challenged due to the independent nature of practice and limited administrative support or ability to find coverage for their patients to participate at planning tables. Time spent at planning tables also means less time with patients, and often a loss of income [15]. Coordination among FPs remains a challenge due to busy practices that are operating in different locations, and minimal networks to connect them or allow for information sharing. As a result, FPs remain the least likely to fully participate in efforts towards healthcare system integration due to the absence of a well-functioning collaboration model (or structure). Pockets of excellence do exist, providing evidence that primary care integration with other healthcare services, and led by FPs, can positively impact population health [16, 17]. It is therefore important to identify existing strategies, processes, and structures that independent FPs are already using to effectively participate in health system integration.

The goal of this review was to better understand the factors that can enable functional structures of collaboration and effective processes for FPs to contribute to health system reform. Our review focused on the structures, or models for collective action, and the processes used by FPs which support their active participation in integrated health care around the globe. Secondarily, we sought to understand the factors of primary care integration through the theoretical models presented as context for such collaborations.

## Methods

We conducted a preliminary search of databases (PROSPERO, MEDLINE, the Cochrane Database of Systematic Reviews and the Joanna Briggs Institute (JBI) Evidence Synthesis) about structures and processes used by FPs/general practitioners (GPs) to support their active collaboration and participation in integrated health care. No current or existing scoping reviews or systematic reviews on this topic were identified.

The framework of this scoping review is based on the latest JBI methodological guidance for the conduct of scoping reviews [18]. We developed steps to identify relevant literature, develop search strategy and inclusion criteria, screen and select studies, and chart and report data. We used Preferred Reporting Items for Systematic Review Meta-Analysis for Scoping Review (PRISMA_ScR) flow diagram to present the search results [18, 19].

This review aims to answer two questions:

1. What structures or processes to build collaboration in primary care are described in the literature?
2. What frameworks/approaches were used in the literature to describe such collaborations?

### Identifying, searching, and selecting relevant studies

We targeted qualitative studies (including those embedded in mixed method studies) that describe structures or models for collaboration and/or processes which further integrated health systems and are considered to be long-term partnerships between FPs/GPs and with other clinical sectors. Relevant literature includes articles that focus on collaborative efforts, formed partnerships, coalitions, alliances, and processes that enable intra-organizational functioning. The sources of relevant literature include published and grey literature; all languages are considered with the help of DeepL (DeepL SE, Cologne, Germany), a translation software capable of translating documents. Broadly, grey literature includes text and opinion papers, theses and dissertations, government reports, organization/association reports, and conference proceedings. Considering that collaboration between FPs and other medical sectors or organizations in integrated health care is a relatively recent concept, the publication time for our review is limited from the year 2000 to 2021.

In consultation with a health sciences librarian, we used a 3-step search strategy to identify relevant articles [18, 19]. First, we performed an initial limited search of MEDLINE database to identify the articles on the topic. We then identified the text words contained in the titles and abstracts and the index terms used to describe the articles (see Table 1).

**Table 1:** Preliminary key words and index terms for search and inclusion criteria

Phenomenon	Population	Study design
Partnership Alliance Inter-organization Coalition Collaborat* Integrat* Cooperat* Associate*	Family doctor Family physician General practitioner	Qualitative study Case study Phenomenology Ethnography Grounded theory Action research
Inclusion criteria		
- Study describing collaborative work for inter-organizational partnerships to further integrated care involving FPs/GPs and working in primary care settings - Study on integrated care with involvement of independent FPs/GPs regardless of their associated payment model and organizational structure - Qualitative study design (phenomenology, grounded theory, ethnography, action research, and case study) - Mixed-methods research in which the qualitative study meets the criteria - Systematic reviews that meet the criteria - Grey literature: ◦ Reports, opinion papers, theses on health team collaborations involving FPs ◦ Frameworks, structures, and/or processes for FPs to engage in inter-organizational teams - Study/document published since 2000 to 2021 - Study/documents published in all languages		
Exclusion criteria		
- Study/documents describing collaborative work within organizations or intra-organizational teams (i.e., multi-disciplinary within a health team) - Study with no involvement of FPs/GPs - Study with nurse-practitioner-led clinics - Quantitative study design - Study/document published prior to 2000		

*Used as wildcard to retrieve variations of term

After using these key words and index terms to perform another search on MEDLINE, we modified and refined some keywords and index, and set up a full search strategy (see Additional file 1: Appendix 1), which we used to perform searches across all included databases: MEDLINE, EMBASE, and Business Source Premier (EBSCOhost). Additionally, the reference lists of included reports and articles were also searched for additional studies.

A search of grey literature was conducted using a four-step process that includes Google, targeted website and targeted database searches, and asking content experts [20]. Grey literature refers to literature not published by traditional means (e.g., academic journals). The search strategy, including relevant search terms, was adapted for each website and database.

**Fig. 1:**
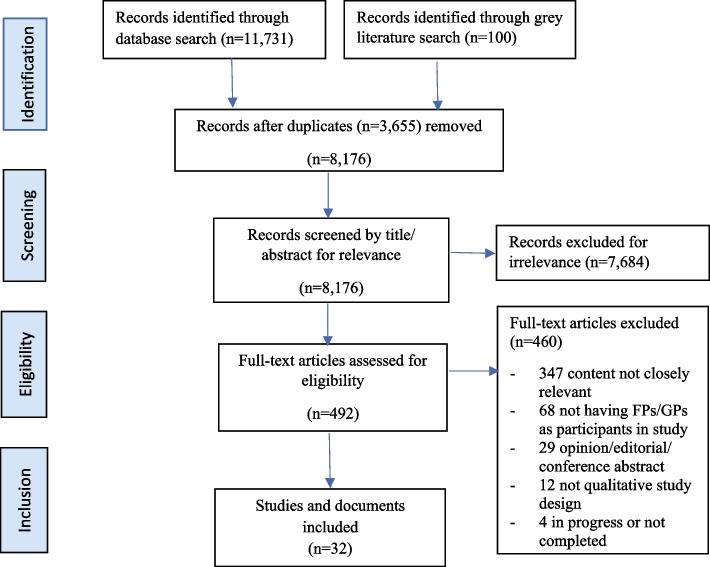
PRISMA-ScR flow diagram for study selection

Articles were selected using the inclusion criteria listed in Table 1. Peer-reviewed study selection followed a two-stage assessment: text/abstract screening and full text review. Each title and abstract were assessed by two independent reviewers from a pool of five reviewers (CG, HH, NA, DHK, and ACB). The full text of selected citations was assessed in detail against the inclusion criteria by two independent reviewers from a pool of four reviewers (CG, HH, ACB, DHK). Reasons for exclusion of full-text papers that did not meet the inclusion criteria were recorded. Any disagreements that arose between reviewers at each stage of the selection process were resolved through discussion or with a third reviewer. The results of the search were reported in the final scoping review and presented in a Preferred Reporting Items for Systematic Reviews and Meta-analyses for Scoping Reviews (PRISMA-ScR) flow diagram (see Fig. 1).

### Charting the data

A data extraction tool was designed in accordance with JBI methodology [18] and modified as necessary during the process of extracting data from each included paper (see Additional file 1: Appendix 2). The variables extracted from selected articles and documents included title, year of publication, origin/country of the study, aim/purpose, study population/sample size, methods, key findings, limitations, future recommendations. Data were extracted from each peer-reviewed or grey literature article by two independent and blinded reviewers from a pool of four reviewers (CG, HH, DHK, and ACB). Disagreements were resolved through discussion or with a third reviewer.

### Collating, summarizing, and reporting the results

A thematic analysis [21] was conducted with the key findings of selected articles and documents. One reviewer (HH) read the key findings thoroughly and identified the major activities of collaboration between FPs/GPs and other clinical sectors for integrated health care. These major activities were used as an initial list of codes, such as ‘communication’, ‘shared vision and values’, and ‘relationship building’. Two reviewers (CG, HH) independently coded the content of the key findings by using the initial codes, and identified new and emerging codes. The reviewers compared the codes and themes, clustered the recurrent themes, identified patterns and relationships between the themes, and organized the themes into interrelated categories. The data are presented in tabular form (see Tables 2, 3 and 4). Also, a narrative summary describes how the results are related to the scoping review questions.

**Table 2:** Identified key factors for building structures for collaboration between family physicians and other healthcare sectors

Factors	Enabling the structure of collaboration	Sources
***1. Shared vision, values, goals***		
* Use a MOU to define common values and vision*	*Have MOUs and value statement, define shared identity and common values, compel a shared vision for change especially around potential benefits for patients, and respect multiple views, and ensure diverse viewpoints can be heard and appreciated*	[22–30]
* Establish shared objectives and goals*	*Identify a common health or organizational goal that is unable to be achieved alone, allow collective goal-setting, and develop consensus on objectives* *Have common agenda, including a common understanding of the problems and a joint approach to solving it through agreed-upon actions*	[25, 26, 28, 30–33]
* Clarify mutual benefit from collaboration*	*Consider the benefits of working together instead of recruiting external help* *Leverage partnership to apply for funding*	[25, 29, 31]
* Develop district health profile*	*Use data from public health, health centres, primary care, and municipality to develop district health profile to establish collective goals*	[34]
***2. Collaborative Leadership***		
* Build strong primary care physician leadership*	*Establish a physician group structure and develop communication channels within the group; build strong physician leadership that can support quality in primary care* *Set up professional committees, communities of practice*	[28, 35–38]
* Establish leadership with broad representation*	*Establish collaborative leadership (i.e., committees, boards), have broad participation and representation to facilitate joint planning, build relationships, guide and coordinate processes, systems thinking, and outcomes* *Establish steering committee that includes community members; related sectors (e.g., primary care, hospitals, *etc*.) send one or two members to higher level board so that each sector is represented and has a voice*	[25, 27, 28, 30, 35, 36, 39]
* Use leadership to create clarity and foster trust*	*Create clarity: vision and mobilization; assess the environment* *Foster trust and good working relationships between collaboration partners, share power and influence* *Contribute to self-growth of group members and regularly engage in self-reflection*	[26, 28]
***3. Collaborative decision-making***		
* Develop a structure to guide and manage collaboration and anchor in primary care*	*Establish an appropriate management structure to execute the leadership team’s vision of the integrated care team. Have common guidelines for new models of collaboration. Use a 'linking organization' that connects actors per issue* *OHTs (Integrated care) should be anchored in primary care and build physician leadership. Formally plan and structure collaboration to guide interactions between physicians and specialists* *Identify appropriate change management tools and resources required to facilitate collaboration across partner organizations*	[27, 29, 35, 37, 39–41]
* Free up resources to be shared among collaboration partners*	*Free up resources to be shared across your system, share project resources, use shared EMR and appointment system, and keep transparency about cost*	[29, 39, 42]
* Formalize communication mechanisms; increase transparency*	*Formalize agreements and regular meetings, have more structured communication such as shared electronic patient records, use common language, use an effective information sharing technique, have regular inter-organizational knowledge sharing* *Keep the patient voice respected*	[25, 27, 32, 36, 37, 39, 42–45]
* Manage organizational stability*	*Develop methods to support network coherence and stability, facilitate relationships through pairing of partners*	[23, 25, 42]
*Develop shared decision- making agreement*	*Develop a governance model or framework for a transparent and constructive approach for decision-making that allow members to hold each other accountable* *Collaborative decision-making agreements need to describe performance management, information management and sharing, resource allocation, conflict resolution, and the extent to which new members can be accommodated*	[27, 30, 45, 46]

**Table 3:** Most effective processes to support collaboration between FPs and other health sectors

Factors	Enabling the processes of collaboration	Sources
***1. Effective Communication***		
* Develop reciprocal communication*	*Primary care physicians and collaboration partners are engaged in purposeful conversation and deliver feedback to each other*	[23, 24, 47, 48]
* Leverage appropriate communication tools and styles*	*Use various tools for effective communication, embrace various communication styles to fit for individual and group communication needs*	[26, 27, 32, 33, 38, 42]
* Work towards continuous, consistent, open communication*	*Communication among partners is continuous, consistent, and open for transparency and sharing of information of patient’s care, and issues and problems*	[24, 27, 29, 30, 33, 36, 38]
* Utilize in-person communication*	*Face-to-face meeting is effective for interpersonal communication; in-person initial meetings are important for multi-sectoral/organizational collaboration on integrated care initiative*	[38, 40, 43, 47, 48]
* Encourage discourse with the communities that are being served*	*Have community or local policy dialogue; create opportunities for learning from external experts and organizations*	[34, 36]
***2. Building relationships***		
* Develop professional and interpersonal relationship between partners*	*Initiate relationships. Knowing one another (e.g., doctor knows pharmacist), such mutual acquaintanceship is a major component of positive experiences that assist collaboration* *Take time to learn about one another to optimize contact and enhance relationships* *Nurture relationship. Build effective professional and interpersonal relationships with providers and other members of each individual patient’s healthcare team*	[32, 33, 36, 42, 49–51]
* Develop a culture of mutual trust*	*Develop a culture of trust across all levels of stakeholders; building trust among partners is a key to relationship building and collaboration between team members*	[29, 30, 33, 36, 40, 45, 49, 52]
* Demonstrate mutual respect*	*Appreciate representation at table from appropriate participants, demonstrate professional respect between professionals, pay attention to language, and spend time on each other’s roles to build capacity of group with added knowledge*	[23, 38, 40, 49, 53]
* Clarify roles, responsibilities, expectations*	*Clarify roles, responsibilities, and expectations by using a MOU (i.e., how do we come to the table together, how are decisions made, what decisions the table can take). Ensure agreed-upon principles and roles, have clearly designed roles and tasks*	[36, 38, 40, 52, 53]
* Share power*	*Share and balance power, share leadership, flatten the hierarchy, and create a safe environment to ask questions*	[24, 28, 32, 40, 52]
***3. Motivation for change***		
* Identify motivation for collaboration*	*Identify motives for collaboration from regular care experiences (patient interest, developing personal relationships, gaining mutual respect); identify motives for the development of new models*	[41, 45]
* Maintain receptivity to novel initiatives*	*Establish a growth mindset, open to new ideas* *Create space to try new initiatives and push existing boundaries. Encourage experimentation while managing risk. Identify appropriate change management tools and resources required to facilitate collaboration across partner organizations*	[23, 24, 27, 37]

**Table 4:** Theoretical frameworks used to explore FP collaboration in health system integration

Theoretical/conceptual framework	Fundamental concepts	Source
*Social Identity Approach (SIA)*	*Viewing self as part of a group, aligning social identity with other members*	[22]
*Framework of interprofessional collaboration*	*Focus on the interaction between the organizational dimensions* *Organizational relationship based on four dimensions – organization, procedural, relational, contextual* *Considers aspects of structure and relationships between individuals’*	[32]
*Competing values framework (CVF)*	*Analyzes organizational effectiveness along three competing value dimensions: external-internal, control-flexibility, means-goals*	[31]

During our scoping review, we consulted two family physicians who have practiced in Ontario for 20–30 years with rich knowledge and experience in primary healthcare reform. We shared the preliminary results and discussed how the themes extracted from the included studies made sense to them. Their positive comments confirmed that our data analysis was accurate, and results were meaningful [54, 55].

## Results

### Summary of the included studies (*n* = 32)

After searching MEDLINE, EMBASE and EBSCOhost databases, 11,731 identified peer-reviewed records were collated and uploaded into Covidence (Covidence, Melbourne, Australia), a web-based screening and data extraction tool for authors conducting systematic and scoping reviews. Covidence identified and removed 3,645 duplicates, leaving 8,086 records for title and abstract screening. Grey literature search identified 100 records, in which 9 duplicates were identified and removed, leaving 91 records for title screen and full text review. Thirty-two (32) studies and documents are included in this scoping review: 22 peer-reviewed studies and 10 grey literature documents. The PRISMA-ScR flow diagram illustrates the numbers of records and the reasons of exclusion in title/abstract screening and full text assessment (see Fig. 1).

Over half of the studies and documents (18) were from Canada (56%), 8 (25%) were from Europe, 3 (9.5%) were from the UK, and 3 (9.5%) were from Australia.

**Fig. 2:**
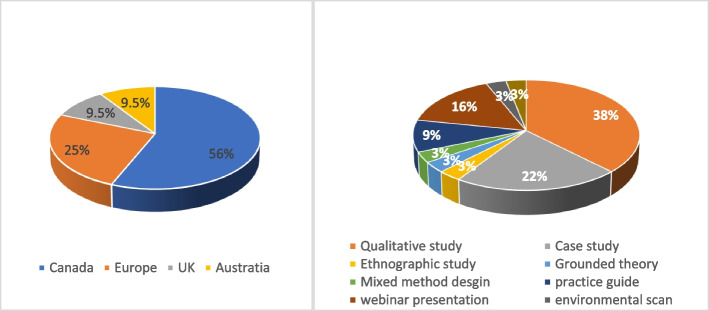
Characteristics of the included studies/documents

Twenty-two (69%) of the included documents were empirical using qualitative research methodology, including qualitative study, case study, ethnographic method, grounded theory, and qualitative study in mixed-method design (see Fig. 2). Data were mostly collected through in-depth semi-structured interviews, focus groups, observations, fieldnotes, document analysis, and survey content analysis.

Grey literature documents (*n* = 10, 31%) were non-empirical studies and include practice guides and toolkits for interprofessional, inter-sectoral collaboration by primary care professional associations (*n* = 3, 10%), webinar presentations on family health teams collaborating with other health organizations in OHTs (*n* = 5, 15%), environmental scan about healthcare inter-organizational collaboration (*n* = 1, 3%), and frameworks for such collaboration (*n* = 1, 3%). Additional file 1: Appendix 3 illustrates details of the characteristics of the included studies and documents.

### Factors deemed most important to any structure and processes for collaboration to enable family physicians’ participation in integrated care

**Fig. 3:**
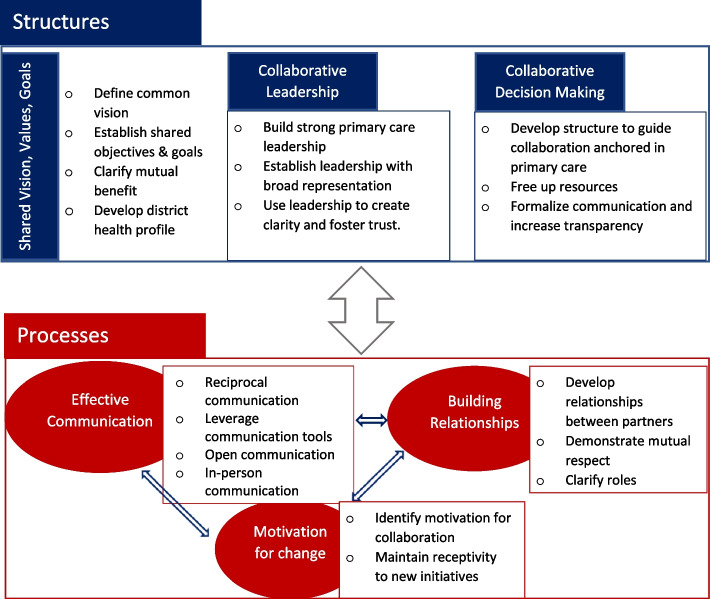
Structure and processes that enable FPs to collaborate with other healthcare sectors in integrated care

This scoping review identifies three main factors related to a structure or model, as well as three main themes related to the processes that are critical to enable collaboration among and with FPs in integrated care (see Fig. 3).

#### Structural factors for integrated care between FPs and other health sectors

Structural factors that enable collaboration among and with FPs in integrated care include: (1) shared vision, values, and goals, (2) collaborative leadership, and (3) collaborative governance for decision-making process.

### Shared vision, values, and goals

A shared vision, and defined values and goals were critical for FPs and other healthcare sectors to collaborate (Table 2). Developing a Memorandum of Understanding (MOU) to clarify common values and vision and identifying goals for change was identified as a key factor to success [22–25]. In addition to clarifying common vision and goals, parties involved also felt it critical to define their shared identity and values [22–30]. One study conducted in Holland [34] reported using data collected from local health and municipal organizations (public health, health centres, primary care, municipality) to develop a district health profile, which guided stakeholders in developing their common vision and goals for collaboration. Common goals clarified the mutual benefits from collaboration, such as sharing resources and working together to apply for funding support [25, 29, 31]. Common goals also included a common understanding of the problems and a joint approach to problem-solving through agreed-upon actions [25, 26, 28, 30, 32, 33].

### Collaborative leadership

Effective collaborative leadership included broad representation from all partners and stakeholders. The leadership structure often took the form of steering committees or boards that included representatives from primary care, hospital, community and municipal, so that the voice of each sector could be heard [25, 27, 28, 30, 35, 36, 39]. Key to the collaborative leadership was strong primary care physician leadership, such as a physician group or professional committee that would develop communication channels within the group and support quality of care [28, 35–37].

The collaborative leadership facilitated joint planning, relationship building, systems thinking, and coordinating collaborative processes [25, 27, 28, 30, 35, 36, 39]. Additionally, leadership was found to play an important role in clarifying shared vision, goals, and values, fostering trust between partners, and promoting personal and organizational growth through collaboration [26, 28].

### Collaborative decision-making

Inter-organizational collaboration requires a framework to guide decision-making, which increases partner accountability. Governance should be fair, formalized, and anchored in primary care. An appropriate management structure could help to execute the leadership team’s vision of the integrated care team, and plan and structure collaboration between primary care physicians and specialists [40]. Additionally, this management structure identified appropriate change management tools and resources to facilitate collaboration across partner organizations [27, 29, 35, 37, 39–41, 45]. Collaboration teams formalized communication methods (i.e., regular meetings, structured types of communication, use of common language) [25, 27, 32, 36–39, 42–45], freed up resources across partners (i.e., use of shared EMR and appointment system, transparency about cost) [29, 39, 42], managed organizational coherence and stability [23, 25, 42], and developed shared decision-making agreements to accommodate diverse viewpoints including conflict resolution for all partners [27, 30, 46].

#### Processes of collaboration for integrated care between FPs and other health sectors

Three main themes emerged related to activities that encouraged FPs’ successful collaboration with other healthcare sectors: (1) effective communication, (2) building relationships, and (3) motivation for change. Table 3 illustrates the main and specific processes of FPs in collaboration with other healthcare sectors in integrated care.

### Effective communication

Developing effective communication was reported in most of the studies and documents. The studies included in this review identified specific processes that made communication effective. Partners engaged in reciprocal communication [23, 24, 47, 49], during which primary care physicians and collaboration partners delivered feedback to each other. They chose appropriate communication tools (e.g., telephone, email, EMR message) and styles (e.g., face-to-face, virtual) to fit both individual and group needs [26, 27, 32, 33, 38, 42]. In-person and face-to-face meetings proved effective for interpersonal and inter-organizational communication [40, 43, 47, 48]. Additionally, partners worked towards continuous, consistent, and open communication to achieve effective patient care, address problems, and find solutions [24, 27, 29, 30, 33, 36, 38].

### Building relationships

Professional and interpersonal relationships between partners were developed from initial face-to-face meetings as well as prior mutual acquaintanceship. Taking time to learn about one another facilitated and supported relationships [32, 33, 36, 42, 49–51]. Developing a culture of mutual trust and respect among partners was key to relationship building between team members [29, 30, 33, 36, 38, 40, 45, 49, 52]. To facilitate trust and respect, partners learned about each other’s roles from various sectors and worked to clarify roles, responsibilities and expectations from all team members [23, 38, 40, 49, 53]. Partners often used MOU to design tasks and ensure agreed-upon roles and principles [36, 40, 52, 53]. Shared power enabled the elimination of a hierarchy creating a safe space for exploring questions [24, 28, 32, 40, 52].

### Motivation for change

Making change in order to improve patient care was a key motivation for FPs to collaborate among themselves and with others. Identifying and highlighting the motivation for change allowed FPs and partners to explore and experiment on new models of care [42]. Being open to new ideas requires a growth mindset [24, 37]. Change management and tools required to support collaboration are needed with an ability to remain motivated to push boundaries to the envisioned change [23, 27, 45].

### Theoretical frameworks/approaches used to understand collaboration between FPs and other health sectors

Three frameworks were identified in the literature that provide insight into collaborative initiatives which included FPs (see Table 4). A Social Identity Approach (SIA) provides a valuable lens that emphasizes the importance of shared identity, especially for FPs/GPs that function autonomously, in achieving change [22] in the UK. Analysis of collaborative initiatives in Belgium using a framework of interprofessional collaboration considers the interactive elements within organizations which can support, or derail, change efforts [32]. Similarly, a study in Australia using a competing values framework (CVF) to support the analysis of collaboration by and with FPs explores dimensions that are seen to be in direct opposition and can create challenges to change efforts if all values must be satisfied [31].

## Discussion

This study provides evidence of change around the globe where FPs take on a central role in collaborative ventures to further the integration of healthcare systems.. Despite the identified challenges to the inclusion of FPs in shaping integrated health systems, our results show that these instances of change are built not just on collaborative efforts but galvanized by key elements related to structures and processes that strengthen change initiatives. A scoping review was chosen to address the research questions due to it breadth and rigorous methodological approach. In this way, this review has met the objective to better understand which structural and processual factors are deemed most important to successful collaboration between FPs and one another, and between FPs and other healthcare sectors. Additionally, this review points to some theoretical frameworks that are useful in understanding factors for consideration which may impact change efforts.

### Structural success factors

To enable FPs’ participation in change initiatives, our results indicate that any structure used to enhance group cohesion and shared decision-making benefited by defining collaboration in advance. The criticality of having a common vision and well-articulated goals aligned with a functional structure is evident. In his seminal work of *Leading Change,* John Kotter pointed out that developing a common vision is one of the fundamental first steps to enacting change and solidifies membership in a shared direction [56]. This is particularly important for health system integration, in which FPs need to be seen as part of a large sector (primary care) rather than from the perspective of their individual practices.

Partnerships or alliances can only be effective when authentic participation is included, particularly the engagement of independent FPs. When healthcare system change initiatives are rooted in primary care, collective decision-making can mitigate the challenges of engaging FPs in integrated health system development. In integrated care initiatives, FPs have an important role to play in collaborative leadership and decision-making. FP leaders are seen as trustworthy and can use their authentic knowledge of the sector to propel change forward.

### Processes that foster inter-organizational collaboration

Two factors are critical in any collaboration: communication that works well, and relationship development. Several of the included studies identified that consistent and open communication coupled with varied tools and methods enabled collaboration. A dedicated and concerted approach to effective communication is particularly important to ensure that information flows to FPs that function independently and in various locations. Just as critical is a focus on allowing for information flow that goes in both directions.

A significant number of studies flagged relationship development as pivotal to success. Capitalizing on existing relationships within primary care, where trust already exists, proves to be an effective way to engage FPs in change efforts. Intentional relationship-building fosters mutual respect and trust which can lead to effective collaboration and successful outcomes. The absence of trust, identified by Lencioni [57] as the first and fundamental reason why teams do not function well thwarts progress.

A lesser number of studies reported that sustaining momentum was an important process which fostered collaboration efforts but was viewed as critical by the authors as change initiatives are known to lose energy over time. Not only was it evident that a growth mindset was important among all parties to remain open to new ideas and possibilities, but also that retaining the vision of change and reasons for change was a motivating factor that propelled projects forward.

### From micro to meso level

In the past two decades, collaboration between FPs and other healthcare professionals was primarily studied from the interprofessional perspective, or at the micro level. Some studies reported on the challenges to primary care providers such as the nature of leadership, shared vision and purposes, and decision-making processes [58]. The evidence identified in this scoping review illustrates that the integration of services requires FPs’ collaboration at both micro and meso levels—the interprofessional level and inter-organizational or inter-sectoral level.

### Limitations

While this scoping review aimed to identify the enablers of both structures for collaboration and processes to support FPs’ participation in health system integration, FPs’ payment models were not included as a factor that may have an impact on their participation. Additionally, as the studies included mainly reported on successful and effective strategies for collaboration among FPs/GPs and other healthcare organizations, the supports identified as lacking (i.e., time away from patients, lack of administrative support, loss of funding) were not addressed in most of the studies, which limits our knowledge of how FPs compensated for these challenges.

### Implications and recommendations

There is an urgency to accelerate efforts around the world in building fully integrated health systems. There is also evidence that supports FPs contribution to change efforts due to the pivotal and primary role they have in healthcare. Intentional and purposeful effort to ensure that FPs are engaged in health system integration is necessary. The best practices and key factors illuminated by this review can guide successful collaborations to achieve this goal.

While FPs may be unified in their experience and knowledge related to patient care, their typically independent nature of practice can impact their ability to identify as part of a larger group. This review identified several key structural factors, processes and frameworks that could be used to support the involvement of FPs in the development of an integrated health system.

These results can inform OHTs as they are strongly focused on primary care engagement. As well, this review can also provide information for health system integration in both a national and international context.

Further research on policies or supports for FPs by government to allow for a sustained approach to their participation and to address the limitations for FPs in contributing their worthy voice to influence change would be a valuable next step from this review.

## Conclusion

The end goal for an integrated healthcare system is that patients receive the care they need seamlessly, with minimal disruption when transitioning between services and providers and that all health care providers and organizations are working together to make this happen. We know that system integration is important, FP knowledge is critical, and that patients will continue to experience delays in receiving coordinated and much-needed care in a system characterized by siloed services.

This in-depth analysis provides some key learnings from successful collaborations around the globe where FPs/GPs were integral to the evolution of integrated health systems. Integrated health systems are not easily achieved as it requires careful planning to break down silos between services that are historically funded and operating separately. Primary care-led systems require even greater energy to achieve due to the disjointedness between most FPs with minimal structures to bring them together and inconsistency in processes that enable communication, joint advocacy and primary care leadership in health system reform.

## Supplementary Information

**Additional file 1: Appendix 1. **Full search strategy.** Appendix 2.  **Data charting protocol.** Appendix 3.** Characteristics of the included studies and documents.

## Data Availability

The datasets used and analysed for this scoping review are available from the corresponding author on request.
