# Supplementary material for: Family physicians collaborating for health system integration: a scoping review
Source: BMC Health Serv Res. 2023 Jan 23;23:68. doi: 10.1186/s12913-023-09063-w (PMC9869511; doi:10.1186/s12913-023-09063-w)
Supplement: Supplementary file 1 — Additional file 1: Appendix 1. Full search strategy. Appendix 2. Data charting protocol. Appendix 3. Characteristics of the included studies and documents. [file 12913_2023_9063_MOESM1_ESM.docx]

**Appendix 1** Full search strategy

| collaborat*.mp. OR intersectoral collaboration/ OR intersectoral cooperation*.mp. OR inter-organization.mp. OR collectivism.mp. OR "Delivery of Health Care, Integrated"/ OR integrated delivery.mp. OR models, organizational/ OR partnership*.mp. OR integrated health*.mp. OR integrated system*.mp. OR intersectoral collaboration*.mp. OR organizational model*.mp. OR alliance*.mp. OR exp Health Care Coalitions/ OR healthcare coalition*.mp. OR health care coalition*.mp. |
| --- |
| AND |
| primary health care/ OR primary care.mp. OR primary healthcare.mp. OR primary health care.mp. OR Physicians, Family/ OR family physician*.mp. OR family doctor*.mp. OR Physicians, Primary Care/ OR General Practice/ OR general practi*.mp. OR General Practitioners/ OR Family Practice/ OR family practi*.mp. OR family medicine.mp. OR general medic*.mp. OR "Continuity of Patient Care"/ OR medical home.mp. OR patient navigation.mp. |
| AND |
| exp qualitative research/ OR Interview/ OR qualitative.af. OR ethnological research.mp. OR (theme$ or thematic).mp. OR phenomenol$.af. OR (grounded adj (theor$ or study or studies or research or analys?s)).af. OR (action research or cooperative inquir$ or co operative inquir$ or co-operative inquir$).mp. OR (humanistic or existential or experiential or paradigm$).mp. OR (field adj (study or studies or research)).tw. OR theoretical sampl$.af. OR ((purpos$ adj4 sampl$) or (focus adj group$)).af. OR (account or accounts or unstructured or open-ended or open ended or text$ or narrative$).mp. OR (life world or life-world or conversation analys?s or personal experience$ or theoretical saturation).mp. OR ((lived or life) adj experience$).mp. OR cluster sampl$.mp. OR observational method$.af. OR content analysis.af. OR (constant adj (comparative or comparison)).af. OR ((discourse$ or discurs$) adj3 analys?s).tw. OR narrative analys?s.af. |

**Appendix 2** Data charting protocol

| Title |  |
| --- | --- |
| Year of publication |  |
| Origin/country (where study was conducted) |  |
| Aims/Purposes |  |
| Study population and sample size |  |
| Methodology/Methods |  |
| Key findings |  |
| Theoretical frameworks/approaches that describe family physician-other health sector collaboration |  |
| Limitations |  |
| Future recommendations |  |

**Appendix 3** Characteristics of the included studies and documents

| Total number of included studies: N=32 |  |
| --- | --- |
| Country/Region | N (%) |
| Canada | 18 (56%) |
| Europe | 8 (25%) |
| UK | 3 (9.5%) |
| Australia | 3 (9.5%) |
| Years of Publication | N (%) |
| 2006-2010 | 1 (3%) |
| 2011-2015 | 11 (36%) |
| 2016-2020 | 20 (61%) |
| Type of Study | N (%) |
| Empirical (qualitative methodology) | 22 (69%) |
| Qualitative study | 12 (38%) |
| Case study | 7 (22%) |
| Ethnographic study | 1 (3%) |
| Grounded theory | 1 (3%) |
| Mixed method design (qualitative component) | 1 (3%) |
| Non-empirical | 10 (31%) |
| Practice guide/tool kit | 3 (10%) |
| Webinar presentation | 5 (15%) |
| Environmental scan | 1 (3%) |
| Framework | 1 (3%) |
